# How to Enjoy the Tooth-Ache

**Published:** 1864-04

**Authors:** 


How to enjoy the Tooth-ache.—To enjoy this delectable pain to its fullest extent, you should have it in all its glory for a week. Let the pain permeate and insinuate into every portion of the diseased member, racing, jumping and springing around generally like rats in a corn-crib; let it ache till yon can't tell whether the pain is in your mouth, on the top of your head, or in your cravat, but rather think it is all around there; let it ache until you feel like it would be a great relief to hold your head up by the ears, and shake out every molar, incisor, grinder and acher in it; let it ache until you are doubtful whether you stand in the position assigned you, or with your heels in the air; let it ache until you seriously believe every bone, nerve and muscle about your body is full of teeth, and that every tooth is aching on its own hook, and then, when you feel like you have enough pain in your individual mouth to fit out a hospital—when you feel like kicking yourself down stairs—when you are exceedingly anxious to break your neck—then, we repeat, you will begin to realize the tooth-ache.—*Western Home Press.*

